# Estimating the Waning Effectiveness of COVID-19 Vaccines From Population-Level Surveillance Data in Hong Kong

**DOI:** 10.1093/infdis/jiaf207

**Published:** 2025-04-18

**Authors:** Haoling Chen, Xiaotong Huang, Can Wang, Benjamin J Cowling, Tim K Tsang

**Affiliations:** WHO Collaborating Centre for Infectious Disease Epidemiology and Control, School of Public Health, Li Ka Shing Faculty of Medicine, The University of Hong Kong, Hong Kong Special Administrative Region, China; WHO Collaborating Centre for Infectious Disease Epidemiology and Control, School of Public Health, Li Ka Shing Faculty of Medicine, The University of Hong Kong, Hong Kong Special Administrative Region, China; WHO Collaborating Centre for Infectious Disease Epidemiology and Control, School of Public Health, Li Ka Shing Faculty of Medicine, The University of Hong Kong, Hong Kong Special Administrative Region, China; WHO Collaborating Centre for Infectious Disease Epidemiology and Control, School of Public Health, Li Ka Shing Faculty of Medicine, The University of Hong Kong, Hong Kong Special Administrative Region, China; Laboratory of Data Discovery for Health, Hong Kong Science and Technology Park, Hong Kong Special Administrative Region, China; WHO Collaborating Centre for Infectious Disease Epidemiology and Control, School of Public Health, Li Ka Shing Faculty of Medicine, The University of Hong Kong, Hong Kong Special Administrative Region, China

**Keywords:** COVID-19, vaccine effectiveness, population-level surveillance data, infectious disease modeling, Bayesian inference

## Abstract

Assessing the time-varying effectiveness of coronavirus disease 2019 (COVID-19) vaccines is critical to guiding vaccine strategies and public health policies. We developed a Bayesian framework to estimate the waning vaccine effectiveness of various doses of CoronaVac and Comirnaty based on population-level surveillance data. We applied this framework to data on a large Omicron BA.2 epidemic in Hong Kong from January to May 2022. Our results indicated that despite high vaccination rates during this period, high vaccine uptake mitigated but did not prevent rapid spread of infection.

## Teaser

A Bayesian framework was developed to estimate the waning vaccine effectiveness of 2 and 3 doses of CoronaVac and Comirnaty based on population-level surveillance data during Omicron BA.2 epidemic in Hong Kong.

The global coronavirus disease 2019 (COVID-19) pandemic impacted millions of people since 2020. It has significantly burdened health care systems, disrupted economies, and altered the daily lives of people worldwide. In response to the pandemic, Hong Kong adopted a stringent elimination strategy, aiming to completely eradicate the virus through strict public health measures. This approach was effective in controlling the spread of the virus until December 2021. However, Hong Kong faced a severe wave of infections driven by the highly transmissible Omicron BA.2 variant, with a total of over 1 million reported cases from January to May 2022, and 9165 deaths [1].

Vaccination is a crucial strategy in fighting COVID-19 [2]. In Hong Kong, 2 main types of vaccines were made available to the public starting in February 2021: the inactivated CoronaVac (Sinovac Biotech) vaccine and the Comirnaty (BNT162b2, Pfizer-BioNTech) vaccine. However, mutations in variants have led to antigen escape, affecting their vaccine effectiveness (VE). To strengthen immunity, booster doses were introduced in November 2021.

The gold standard to estimate VE is randomized controlled trials; however, these are resource intensive and therefore may not be feasible and ethical, particularly for purpose of monitoring VE. Various methods, such as test-negative design studies, have been used to estimate the waning VE in different countries, but they require comprehensive individual-level data and intensive resources to conduct studies [3, 4]. During public health emergencies such as the COVID-19 pandemic or for the purpose of monitoring VE, using surveillance data to estimate VE offers a practical approach to gauge real-world effectiveness. Although government agencies routinely collect individual-level data, our approach leverages the aggregate summaries that hospitals and clinics can more easily and readily provide. This significantly reduces the data collection burden and alleviates privacy concerns compared to cohort or test-negative studies that rely on comprehensive individual-level information.

In this study, we extended the Bayesian framework of Murayama et al [5] to assess the waning VE of both the ancestral monovalent CoronaVac and Comirnaty vaccines using population-level surveillance data from Hong Kong. We focus on evaluating the waning VE of these vaccines over time, particularly after the administration of 2 and 3 doses.

## METHODS

### Study Design

We aimed to estimate the waning in protection against COVID-19 infection of 2 types of vaccines during the Omicron wave that occurred from January to May 2022 in Hong Kong, which had a population of 7.5 million. Vaccination records were required to be reported. A COVID-19 database was obtained from the Center of Health Protection in Hong Kong, featuring detailed vaccination records and case data connected by unique identifiers during this period. These vaccination records documented the date and type of each administered vaccine dose, including CoronaVac (Sinovac Biotech) and Comirnaty (BNT162b2, Pfizer-BioNTech) vaccines. Hence, time series of cases by vaccination status (types and dose numbers of vaccination), and time series of vaccinations by type and dose number could be constructed.

Individuals who were partially vaccinated, having received only 1 dose of either CoronaVac or Comirnaty, were categorized as unvaccinated in this analysis. Additionally, we assumed that each vaccine dose took 14 days to achieve full effectiveness; thus, infections within 14 days postvaccination were attributed to the preceding dose.

### Statistical Methods

We extended a previously developed framework applied in Japan [5] to estimate waning VE for different types and doses. In this framework, discrete renewal process was constructed to describe the incidence of new infections among populations with different vaccination status in week *t*, under the assumption of proportionate mixing [6, 7]. We defined θ as the type of vaccine and ξ as the number of doses received. We then denoted $V_{\theta ,\xi }$ as the cumulative proportions of the population vaccinated with θ type and ξ dose. Additionally, *i* and $j_{\theta ,\xi }$ represent the weekly number of new infections among the unvaccinated and vaccinated individuals, where $j_{\theta ,\xi }$ refers to those vaccinated with θ type and ξ dose. Hence, the expected number of infections among population with θ -type and ξ -dose vaccination is


(1) $$\begin{array}{rl} E[i(t)] & =(1-(V_{s,2}(t)+V_{b,2}(t)))R_{i}(t)\overset{t-1}{\underset{\tau =1}{\sum }}\;(i(\tau )+j_{s,2}(\tau )\\ & \;+\;j_{b,2}(\tau ))f(t-\tau ), \end{array}$$

(2) $$\begin{array}{c} E[\;j_{\theta ,\xi }(t)]=V_{\theta ,\xi }(t)R_{\theta ,\xi }(t)\overset{t-1}{\underset{\tau =1}{\sum }}\;(i(\tau )+j_{s,2}(\tau )+j_{b,2}(\tau ))f(t-\tau ), \end{array}$$

where E[⋅] is the expectation operator and *f* is the probability mass function for the generation time interval between infections in primary and secondary case pairs. The mean secondary transmissions for these vaccination groups are denoted $R_{i}$ and $\;R_{\theta ,\xi }$, related by the cross-sectional protection $\varepsilon_{\theta ,\xi }$: $\;R_{\theta ,\xi }(t)=(1-\varepsilon_{\theta ,\xi }(t))R_{i}(t).$ This cross-sectional protection, referred to as the vaccine-derived population protection (VPP), reflects the cumulative protection provided by corresponding type of vaccines at the population level during week *t*. It can be calculated using the vaccine-specific waning VE and the vaccination rate:


$$\zeta_{\theta ,\xi }(t)=E[\varepsilon_{\theta ,\xi }(t)]=\overset{t}{\underset{\tau =1}{\sum }}\;v_{\theta ,\xi }(t-\tau )\cdot \omega_{\theta ,\xi }(\tau ),$$

where $v_{\theta ,\xi }$ represent the weekly per capita vaccination rate, and $\omega_{\theta ,\xi }$ denotes the waning VE over time of vaccination group θ,ξ. We employed an exponential decay model to describe each waning VE. This model assumed that the VE decreases at a rate proportional to its current value, leading to a rapid initial decline that gradually slows down. The exponential decay is given by


$$\omega_{\theta ,\xi }(t)=\alpha_{\theta ,\xi }\cdot exp(-\lambda_{\theta ,\xi }(t-1)),$$

where $\alpha_{\theta ,\xi }$ is the initial effectiveness and $\lambda_{\theta ,\xi }$ is the decay constant for vaccine θ,ξ. Hence, the VPP of the population was the sum of the VPP of all vaccine types and doses.

We applied the framework to estimate the waning VE of 2- and 3-dose CoronaVac and Comirnaty vaccines from the week of 31 December 2021 to the week of 28 May 2022. Our inference was based on a Bayesian framework and a Markov Chain Monte Carlo algorithm was used to estimate the model parameters. A sensitivity analysis using logistic waning was conducted (Supplementary Material 1 and Supplementary Figure 1), and simulation studies were performed to validate our approaches (Supplementary Material 2 and Supplementary Figures 2 and 3).

## RESULTS

### Omicron BA.2 Epidemic in Hong Kong

**Figure 1.**
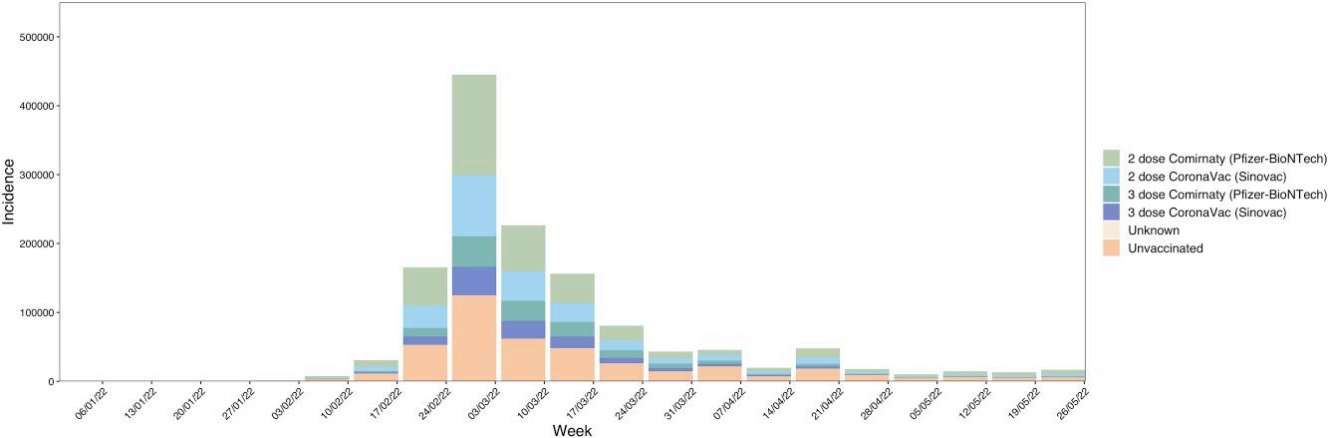
Reported weekly number of COVID-19 cases with vaccine status in Hong Kong.

Before the spread of Omicron BA.2 in Hong Kong (by 31 December 2021), 23% and 2.7% of the population had received 2 doses and 3 doses of CoronaVac, and 39% and 2.5% had received 2 doses and 3 doses of Comirnaty, respectively. From 31 December 2021 to 29 May 2022, there were 1 343 315 reported cases in Hong Kong. Among them, 18.88% and 9.21% received 2 doses and 3 doses of CoronaVac, while 30% and 10.48% received 2 doses and 3 doses of Comirnaty. Case numbers surged from the week of 21 February 2022, peaking at 445 075 reported cases in the week of 28 February 2022 (Figure 1).

**Figure 2.**
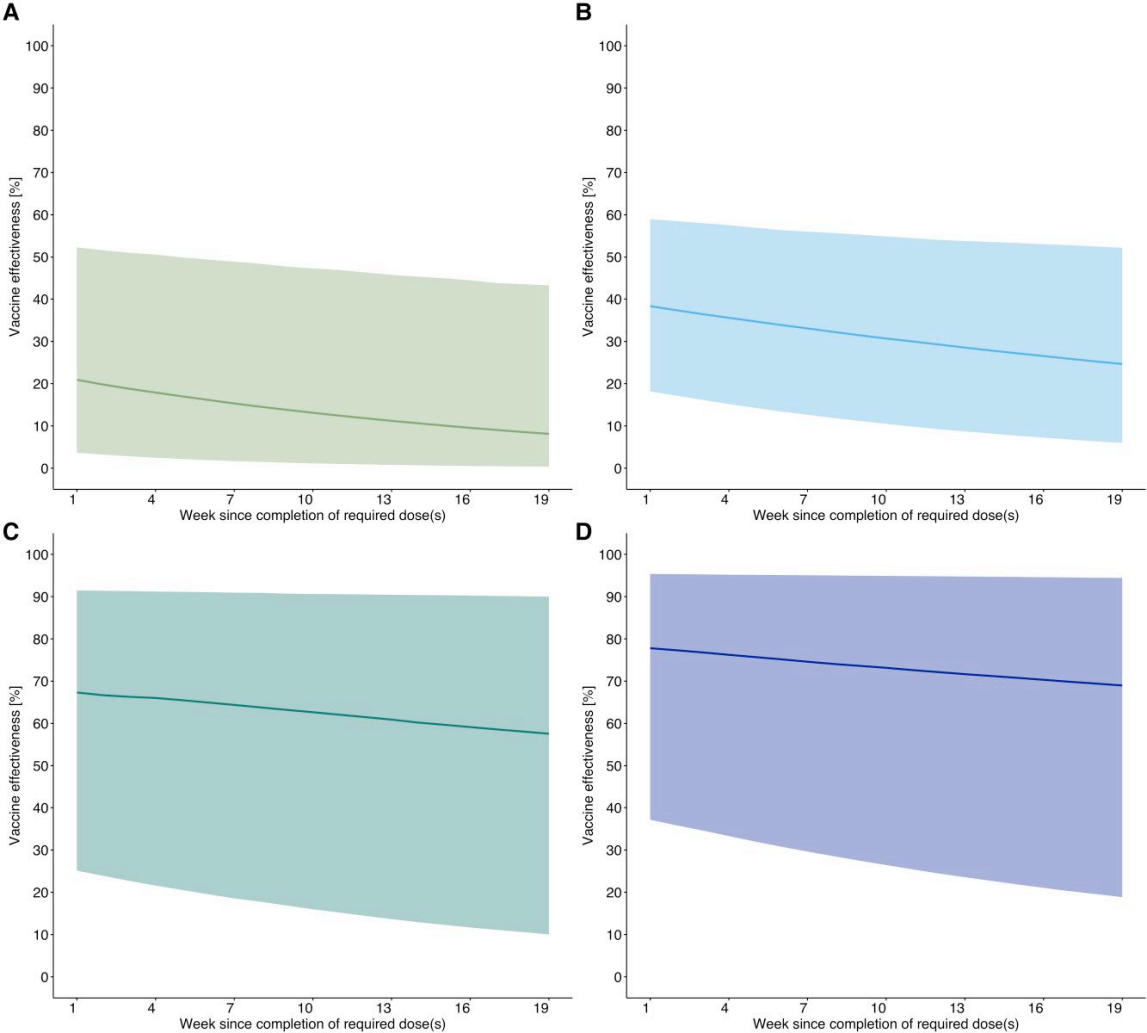
*A*–*D*, The estimated waning vaccine effectiveness using Hong Kong data. The estimated waning effectiveness of (*A*) 2-dose CoronaVac, (*B*) 2-dose Comirnaty, (*C*) 3-dose CoronaVac, and (*D*) 3-dose Comirnaty against Omicron BA.2 in Hong Kong. The lines and shaded areas indicate the estimates and 95% confidence intervals, respectively.

Based on our approach assuming exponential decay of VE, we estimated the initial VE against Omicron BA.2 for 2-dose CoronaVac and Comirnaty were 21% (95% confidence interval [CI], 4%–53%) and 40% (95% CI, 20%–63%); the VE for 3-dose CoronaVac and Comirnaty were 66% (95% CI, 26%–90%) and 77% (95% CI, 37%–95%). After 5 months, the VE for 2-dose CoronaVac and Comirnaty dropped to 1% (95% CI, 0%–47%) and 27% (95% CI, 6%–56%); the VE for 3-dose CoronaVac and Comirnaty dropped to 55% (95% CI, 10%–88%) and 68% (95% CI, 19%–94%). The corresponding weekly waning rates were 5% for 2-dose CoronaVac, 3% for 2-dose Comirnaty, 2% for 3-dose CoronaVac, and 1% for 3-dose Comirnaty (Figure 2).

Based on our model, the VPP was 19% (95% CI, 13%–25%) at the start of the outbreak. Breaking this down by specific vaccine types and dosages, 2-dose CoronaVac provided an VPP of 3% (95% CI, 1%–4%). In contrast, 2-dose Comirnaty started with a 7% VPP (95% CI, 4%–10%). With the 3-dose CoronaVac and Comirnaty, initial VPP was 3% (95% CI, 2%–4%) and 5% (95% CI, 4%–5%), given the low coverage of booster dose.

The estimated number of confirmed cases, based on our calculated VE, aligned with the actual observed cases, demonstrating adequate model fit and robustness (Supplementary Figures 4 and 5).

### Simulated Outbreak Data

We simulated 20 outbreak scenarios consisting of 5 vaccine statuses over time and estimated the waning VE for each vaccine status from the simulated data based on our model. The 95% credible intervals of the estimated waning VE of each vaccine status contained the simulation values, suggesting that our parametric approach yielded adequate posterior distribution of VE for CoronaVac and Comirnaty (Supplementary Figures 2 and 3).

## DISCUSSION

In this study, we estimated the waning VE of 2 widely adopted COVID-19 vaccines, CoronaVac and Comirnaty, during the Omicron BA.2 outbreak in early 2022 in Hong Kong. By analyzing population-level surveillance data with detailed time series of cases by vaccination status and vaccinations by type and dose number, we obtained insights into the VE waning and dynamics of the population-level VE. Although individual-level data were available in this study, our analytical framework operates at the population level, using aggregated time series of cases by vaccination status and vaccine administration data rather than conducting individual-level linkage analyses.

We estimated a rapid decline in 2-dose VE against COVID-19, particularly against the Omicron variant, which aligns with previous studies [8, 9]. The estimated initial VE against Omicron BA.2 was 21% for CoronaVac and 40% for Comirnaty, which was low. Therefore, when the VE waned it was close to zero within a few months to a year, highlighting the importance of booster dose.

Booster doses that included the ancestral strain or ancestral spike mRNA were found to significantly enhance cross-protection against Omicron BA.2 [10]. For individuals receiving a third dose, our estimated initial VE against Omicron BA.2 was higher for both CoronaVac and Comirnaty than reported in other studies [9, 11, 12]; however, their estimates were still covered by the large confidence intervals for our estimates. We estimated that the booster-dose VE remained moderately high during half a year for both CoronaVac and Comirnaty, proving their critical role in maintaining high levels of immunity.

However, immunity after vaccination does clearly wane over time. One reason is linked to a gradual decline in antibody levels [13]. The other reason is due to immune escape associated with the evolution of circulating strains. The waning in VE estimated in our study relates to the former, because Omicron BA.2 was the predominant strain during the entire epidemic studied.

Despite a high individual vaccination rate, our analysis revealed a low (19%) contribution of vaccination to population-level protection in Hong Kong before this Omicron wave. The potential reason was that primary vaccination started in April 2021 in Hong Kong, such that at the beginning of the Omicron BA.2 epidemic in early 2022 many vaccinated people had experienced substantial waning in VE. In addition, the very low cumulative number of infections prior to 2022 resulted in no immunity from natural infection, and thus the whole Hong Kong population was almost completely susceptible [14]. This may explain the high cumulative incidence of infection in this outbreak, which was estimated to be higher than 40% [9].

Our study has some limitations. First, we assumed that individuals with different vaccination statuses mixed proportionately and randomly. Second, our analysis did not consider immunity acquired through natural infection, as we did not model the unreported infections. Third, we did not account for the depletion-of-susceptible bias. This bias is considered minor in the situation reported here [5], but its potential implications should be acknowledged when applying our method to other diseases. Additionally, we did not factor in the variability in waning VE among vaccinated individuals. Finally, given that aggregated data is used, the estimated waning may result in large uncertainty with large confidence interval. Further research should integrate natural immunity, individual behaviors, and vaccination status to estimate the complete profile of population immunity [15].

In conclusion, we developed a Bayesian model to estimate the waning VE of COVID-19 vaccines with multiple types and doses using population-level data. Our approach estimates waning VE using aggregated surveillance data, which is practical when individual-level data are scarce. It simultaneously analyzes multiple vaccine types and dosing regimens, unlike prior studies that focused on a single vaccine. Whereas test-negative and cohort studies require detailed individual-level timing of vaccination and infection, our method only needs time series of cases by vaccination status and vaccine administration records, making it valuable during public health emergencies. Moreover, it explicitly models epidemic dynamics and vaccination history distributions, capturing temporal variations in the force of infection that static approaches may miss. This enables more timely assessments of waning VE and population-level protection during epidemics, which is crucial for evaluation and guidance of ongoing vaccine strategies and policies.

## Supplementary Material

jiaf207_Supplementary_Data

## Supplementary Data

Supplementary materials are available at *The Journal of Infectious Diseases* online (http://jid.oxfordjournals.org/). Supplementary materials consist of data provided by the author that are published to benefit the reader. The posted materials are not copyedited. The contents of all supplementary data are the sole responsibility of the authors. Questions or messages regarding errors should be addressed to the author.
