# Supplementary material for: Estimating the Waning Effectiveness of COVID-19 Vaccines From Population-Level Surveillance Data in Hong Kong
Source: J Infect Dis. 2025 Apr 18;232(2):e341–5. doi: 10.1093/infdis/jiaf207 (PMC12349934; doi:10.1093/infdis/jiaf207)
Supplement: jiaf207_Supplementary_Data [file jiaf207_supplementary_data.pdf]

Supplementary Information

for 'Estimating the waning effectiveness of COVID-19 vaccines from population-level surveillance data in Hong Kong'

Contents

1 Full Bayesian framework 2

2 Simulation study 5

1 Full Bayesian framework

We defined θ as the type of vaccine and ξ as the number of doses received. We then denoted $V_{\theta,\xi}$ as the cumulative proportions of the population vaccinated with θ type and ξ doses. Additionally, i and $j_{\theta,\xi}$ represent the weekly number of new infections among the unvaccinated and vaccinated individuals, where $j_{\theta,\xi}$ specifically refers to those vaccinated with θ type and ξ dose. If an individual has received 2-dose CoronaVac only, they are included in group $j_{s,2}$; if they have received 3-dose CoronaVac, they are included in both groups $j_{s,2}$ and $j_{s,3}$. The same categorization applies to Comirnaty recipients as well. Under proportionate mixing, expected number of infections among population with vaccination θ-type and ξ-dose vaccination is

$$E[i(t)] = \left(1 - \left(V_{s,2}(t) + V_{b,2}(t)\right)\right) R_i(t) \sum_{\tau=1}^{t-1} \left(i(\tau) + j_{s,2}(\tau) + j_{b,2}(\tau)\right) f(t-\tau), \quad (1)$$

$$E\left[j_{\theta,\xi}(t)\right] = V_{\theta,\xi}(t) R_{\theta,\xi}(t) \sum_{\tau=1}^{t-1} \left(i(\tau) + j_{s,2}(\tau) + j_{b,2}(\tau)\right) f(t-\tau), \quad (2)$$

where $E[\cdot]$ is the expectation operator and f is the probability mass function for the generation time interval between infections in primary and secondary case pairs. The mean secondary transmissions for these vaccination groups are denoted R_i and $R_{\theta,\xi}$, related by the cross-sectional protection (VCP) $\varepsilon_{\theta,\xi}$ as follows:

$$R_{\theta,\xi}(\mathrm{t}) = \left(1 - \varepsilon_{\theta,\xi}(t)\right) R_i(t).$$

Each VCP reflects the cumulative protection provided by corresponding type of vaccines at the population level during week t. It can be calculated using the vaccine-specific waning effectiveness and the vaccination rate:

$$\zeta_{\theta,\xi} = E[\varepsilon_{\theta,\xi}(t)] = \sum_{\tau=1}^{t} v_{\theta,\xi}(t-\tau) \cdot \omega_{\theta,\xi}(\tau),$$

where $v_{\theta,\xi}$ represent the weekly per capita vaccination rate, while $\omega_{\theta,\xi}$ denote the waning effectiveness over time of these respective vaccination group θ, ξ. Here, we assumed $\varepsilon_{\theta,\xi}$ follows a beta distribution:

$$\varepsilon_{\theta,\xi} \sim Beta\left(\zeta_{\theta,\xi} \cdot \eta \cdot j_{\theta,\xi}, \quad \eta \cdot j_{\theta,\xi} \cdot (1 - \zeta_{\theta,\xi})\right),$$

where η is the dispersion parameter controlling the variance of $\varepsilon_{\theta,\xi}$, thus we obtained

$$E[\varepsilon_{\theta,\xi}] = \zeta_{\theta,\xi} \text{ and } Var[\varepsilon_{\theta,\xi}] = \zeta_{\theta,\xi} \cdot (1 - \zeta_{\theta,\xi})/(\eta \cdot j_{\theta,\xi}).$$

We employed an exponential decay model to describe each waning VE. This model assumed that the VE decreases at a rate proportional to its current value, leading to a rapid initial decline that gradually slows down. The formula for exponential decay is given by

$$\omega_{\theta,\xi}(t) = \alpha_{\theta,\xi} \cdot exp\left(-\lambda_{\theta,\xi}(t-1)\right),$$

where $\alpha_{\theta,\xi}$ is the initial effectiveness and $\lambda_{\theta,\xi}$ is the decay constant for vaccine θ, ξ.

We also conducted a sensitivity analysis using logistic waning. This approach characterized by an initial phase of rapid decline, which slows down as it approaches a lower asymptote. (Figure S1) The formula for logistic curve is

$$\omega_{\theta,\xi}(t) = 2\alpha_{\theta,\xi} \cdot \left(\frac{1}{1 + exp\left(\lambda_{\theta,\xi}(t-1)\right)}\right).$$

We then formulated the Poisson likelihood for the observed incidence:

$$\begin{aligned}
L(\boldsymbol{Y}_{1:t}|\boldsymbol{\Phi}) = \prod_t & Pois\left(i(t)\middle|E_{i_{1:t-1}, j_{s,2_{1:t-1}}, j_{b,2_{1:t-1}}, j_{s,3_{1:t-1}}, j_{b,3_{1:t-1}}}[i(t)]\right) \\
\times Pois & \left(j_{s,2}(t)\middle|E_{i_{1:t-1}, j_{s,2_{1:t-1}}, j_{b,2_{1:t-1}}, j_{s,3_{1:t-1}}, j_{b,3_{1:t-1}}}[j_{s,2}(t)]\right) \\
& \cdot Beta\left(\varepsilon_{s,2}(t)\middle|\ \zeta_{s,2}(t)\eta j_{s,2}(t),\ \eta j_{s,2}(t)\left(1 - \zeta_{s,2}(t)\right)\right) \\
\times Pois & \left(j_{b,2}(t)\middle|E_{i_{1:t-1}, j_{s,2_{1:t-1}}, j_{b,2_{1:t-1}}, j_{s,3_{1:t-1}}, j_{b,3_{1:t-1}}}[j_{b,2}(t)]\right) \\
& \cdot Beta\left(\varepsilon_{b,2}(t)\middle|\ \zeta_{b,2}(t)\eta j_{b,2}(t),\ \eta j_{b,2}(t)\left(1 - \zeta_{b,2}(t)\right)\right) \\
\times Pois & \left(j_{s,3}(t)\middle|E_{i_{1:t-1}, j_{s,2_{1:t-1}}, j_{b,2_{1:t-1}}, j_{s,3_{1:t-1}}, j_{b,3_{1:t-1}}}[j_{s,3}(t)]\right) \\
& \cdot Beta\left(\varepsilon_{s,3}(t)\middle|\ \zeta_{s,3}(t)\eta j_{s,3}(t),\ \eta j_{s,3}(t)\left(1 - \zeta_{s,3}(t)\right)\right) \\
\times Pois & \left(j_{b,3}(t)\middle|E_{i_{1:t-1}, j_{s,2_{1:t-1}}, j_{b,2_{1:t-1}}, j_{s,3_{1:t-1}}, j_{b,3_{1:t-1}}}[j_{b,3}(t)]\right) \\
& \cdot Beta\left(\varepsilon_{b,3}(t)\middle|\ \zeta_{b,3}(t)\eta j_{b,3}(t),\ \eta j_{b,3}(t)\left(1 - \zeta_{b,3}(t)\right)\right),
\end{aligned}$$

where $\boldsymbol{Y}_{1:t}$ is the observed data and $\boldsymbol{\Phi}$ is the estimated parameters. The detailed

variables and model parameters are outlined in Table S1, whereas the corresponding parameter prior distributions are summarized in Table S2.

2 Simulation study

To validate that our approach could provide unbiased estimates of waning VE for various doses of CoronaVac and Comirnaty, we conducted a simulation study for both exponential and logistic decay model. We simulated 20 outbreak scenarios consisting of five vaccine status over time and estimated the waning VE for each vaccine status from the simulated data based on our model. We set the initial numbers of cases as $i(1,2) = (3500, 3300)$, $j_{\mathrm{s},2}(1,2) = (3,5)$, $j_{\mathrm{b},2}(1,2) = (3,5)$, $j_{\mathrm{s},3}(1,2) = (1,3)$ and $j_{\mathrm{b},3}(1,2) = (1,3)$. Using similar settings in section 1, we tested whether the estimates could be reproduced with different initial values, which might result in various outbreak shapes. The 95% credible intervals of the estimated waning VE of each vaccine status could contain the simulation vales, suggesting that our parametric approach yielded adequate posterior distribution of VE for CoronaVac and Comirnaty. (Figure S2, S3 (A)-(D))

3 Code Availability

All the analysis was conducted in R v.4.2.0. All codes used in analysis with simulated data are available at (https://gitlab.com/haolingchen/hk-covid-ve-analysis).

Supplementary Table

Table S1. Variables and model parameters in the framework.

Parameter	Description	Type
$V_{s,2}(t)$	Cumulative proportion 2-dose CoronaVac-vaccinated per week	Observed
$V_{b,2}(t)$	Cumulative proportion 2-dose Comirnaty-vaccinated per week	Observed
$V_{s,3}(t)$	Cumulative proportion 3-dose CoronaVac-vaccinated per week	Observed
$V_{b,3}(t)$	Cumulative proportion 3-dose Comirnaty-vaccinated per week	Observed
$i(t)$	Number of new infections with unvaccinated status per week	Observed
$j_{s,2}(t)$	Number of new infections with 2-dose CoronaVac-vaccinated status per week	Observed
$j_{b,2}(t)$	Number of new infections with 2-dose Comirnaty-vaccinated status per week	Observed
$j_{s,3}(t)$	Number of new infections with 3-dose CoronaVac-vaccinated status per week	Observed
$j_{b,3}(t)$	Number of new infections with 3-dose Comirnaty-vaccinated status per week	Observed
$R_i(t)$	Mean secondary transmissions in a wholly unvaccinated population	Estimated
$R_{s,2}(t)$	Mean secondary transmissions in a wholly 2-dose CoronaVac-vaccinated population	Latent
$R_{b,2}(t)$	Mean secondary transmissions in a wholly 2-dose Comirnaty-vaccinated population	Latent
$R_{s,3}(t)$	Mean secondary transmissions in a wholly 3-dose CoronaVac-vaccinated population	Latent
$R_{b,3}(t)$	Mean secondary transmissions in a wholly 3-dose Comirnaty-vaccinated population	Latent
$\epsilon_{s,2}(t)$	Vaccine-derived population protection (VPP) for 2-dose CoronaVac-vaccinated	Latent
$\epsilon_{b,2}(t)$	Vaccine-derived population protection (VPP) for 2-dose Comirnaty-vaccinated	Latent
$\epsilon_{s,3}(t)$	Vaccine-derived population protection (VPP) for 3-dose CoronaVac-vaccinated	Latent
$\epsilon_{b,3}(t)$	Vaccine-derived population protection (VPP) for 3-dose Comirnaty-vaccinated	Latent
$v_{s,2}(t)$	Weekly rate of 2-dose CoronaVac vaccination per capita	Observed
$v_{b,2}(t)$	Weekly rate of 2-dose Comirnaty vaccination per capita	Observed
$v_{s,3}(t)$	Weekly rate of 3-dose CoronaVac vaccination per capita	Observed
$v_{b,3}(t)$	Weekly rate of 3-dose Comirnaty vaccination per capita	Observed
$\omega_{s,2}(t)$	Waning vaccine effectiveness of 2-dose CoronaVac over time	Latent
$\omega_{b,2}(t)$	Waning vaccine effectiveness for 2-dose Comirnaty over time	Latent
$\omega_{s,3}(t)$	Waning vaccine effectiveness for 3-dose CoronaVac over time	Latent
$\omega_{b,3}(t)$	Waning vaccine effectiveness for 3-dose Comirnaty over time	Latent
$\alpha_{s,2}$	Initial vaccine effectiveness of 2-dose CoronaVac ($\alpha_{s,2} \geq 0$)	Estimated
$\alpha_{b,2}$	Initial vaccine effectiveness for 2-dose Comirnaty ($\alpha_{b,2} \geq 0$)	Estimated
$\alpha_{s,3}$	Initial vaccine effectiveness for 3-dose CoronaVac ($\alpha_{s,3} \geq 0$)	Estimated
$\alpha_{b,3}$	Initial vaccine effectiveness for 3-dose Comirnaty ($\alpha_{b,3} \geq 0$)	Estimated
$\lambda_{s,2}$	Exponential decay constant for 2-dose CoronaVac ($\lambda_{s,2} \geq 0$)	Estimated
$\lambda_{b,2}$	Exponential decay constant for 2-dose Comirnaty ($\lambda_{b,2} \geq 0$)	Estimated
$\lambda_{s,3}$	Exponential decay constant for 3-dose CoronaVac ($\lambda_{s,3} \geq 0$)	Estimated
$\lambda_{b,3}$	Exponential decay constant for 3-dose Comirnaty ($\lambda_{b,3} \geq 0$)	Estimated
η	Hyperparameter controlling the variance of $\epsilon_{s,2}$, $\epsilon_{b,2}$, $\epsilon_{s,3}$ and $\epsilon_{b,3}$ ($\eta \geq 0$)	Estimated

Table S2. Model parameters and corresponding priors.

Parameter	Description	Priors
$R_i(t)$	Mean secondary transmissions in a wholly unvaccinated population	LeftTruncatedNormal(1, 2)
$\alpha_{s,2}$	Initial vaccine effectiveness of 2-dose CoronaVac ($\alpha_{s,2} \geq 0$)	Uniform(0, 1)
$\alpha_{b,2}$	Initial vaccine effectiveness for 2-dose Comirnaty ($\alpha_{b,2} \geq 0$)	Uniform(0, 1)
$\alpha_{s,3}$	Initial vaccine effectiveness for 3-dose CoronaVac ($\alpha_{s,3} \geq 0$)	Uniform(0, 1)
$\alpha_{b,3}$	Initial vaccine effectiveness for 3-dose Comirnaty ($\alpha_{b,3} \geq 0$)	Uniform(0, 1)
$\lambda_{s,2}$	Exponential decay constant for 2-dose CoronaVac ($\lambda_{s,2} \geq 0$)	HalfNormal(10)
$\lambda_{b,2}$	Exponential decay constant for 2-dose Comirnaty ($\lambda_{b,2} \geq 0$)	HalfNormal(10)
$\lambda_{s,3}$	Exponential decay constant for 3-dose CoronaVac ($\lambda_{s,3} \geq 0$)	HalfNormal(10)
$\lambda_{b,3}$	Exponential decay constant for 3-dose Comirnaty ($\lambda_{b,3} \geq 0$)	HalfNormal(10)
η	Hyperparameter controlling the variance of $\epsilon_{s,2}$, $\epsilon_{b,2}$, $\epsilon_{s,3}$ and $\epsilon_{b,3}$ ($\eta \geq 0$)	HalfNormal(100)

Supplementary Figure.

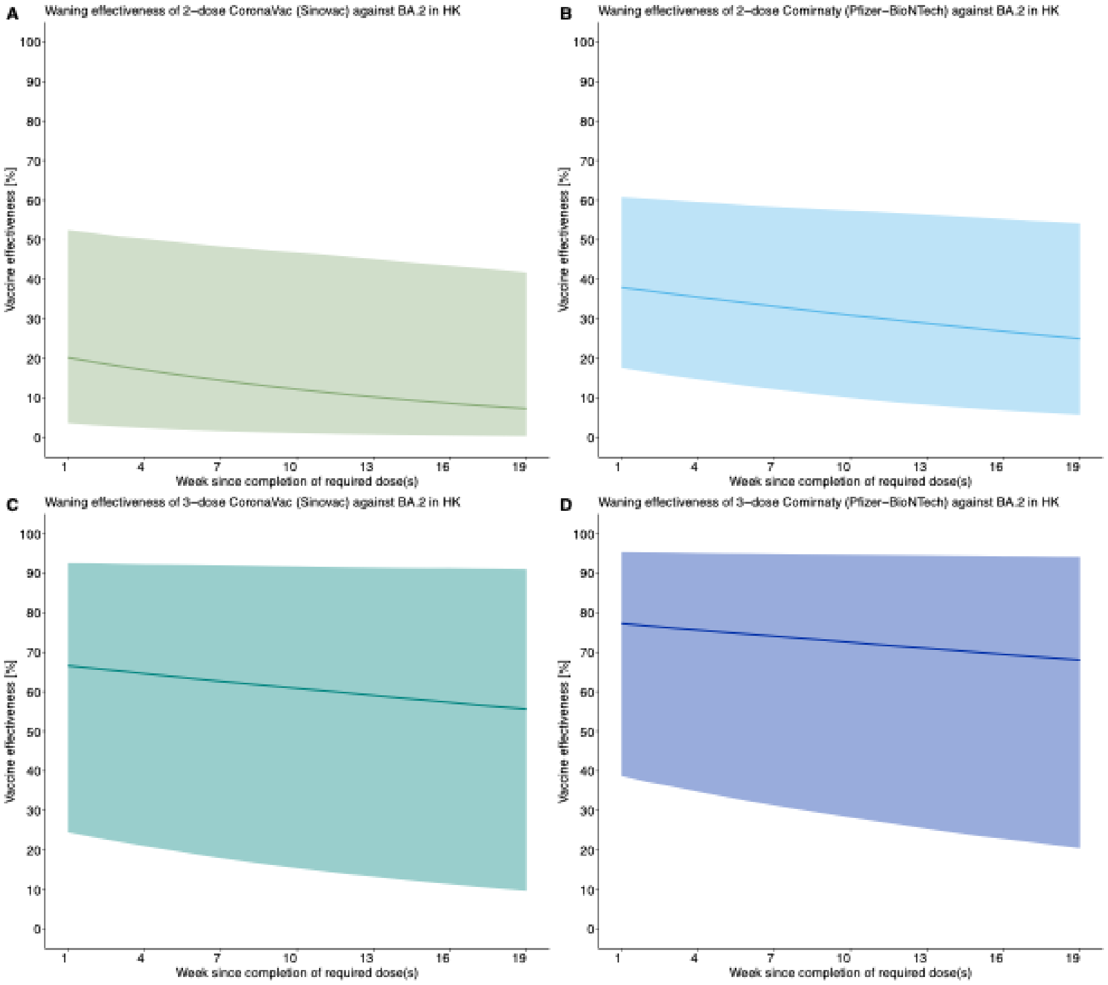

Figure S1. The estimated waning VE through logistic decay using HK data. (A)-(D) The estimated waning effectiveness of CoronaVac and Comirnaty against BA.2 in HK. The lines and shades indicate the medians and 95% confidence intervals, respectively.

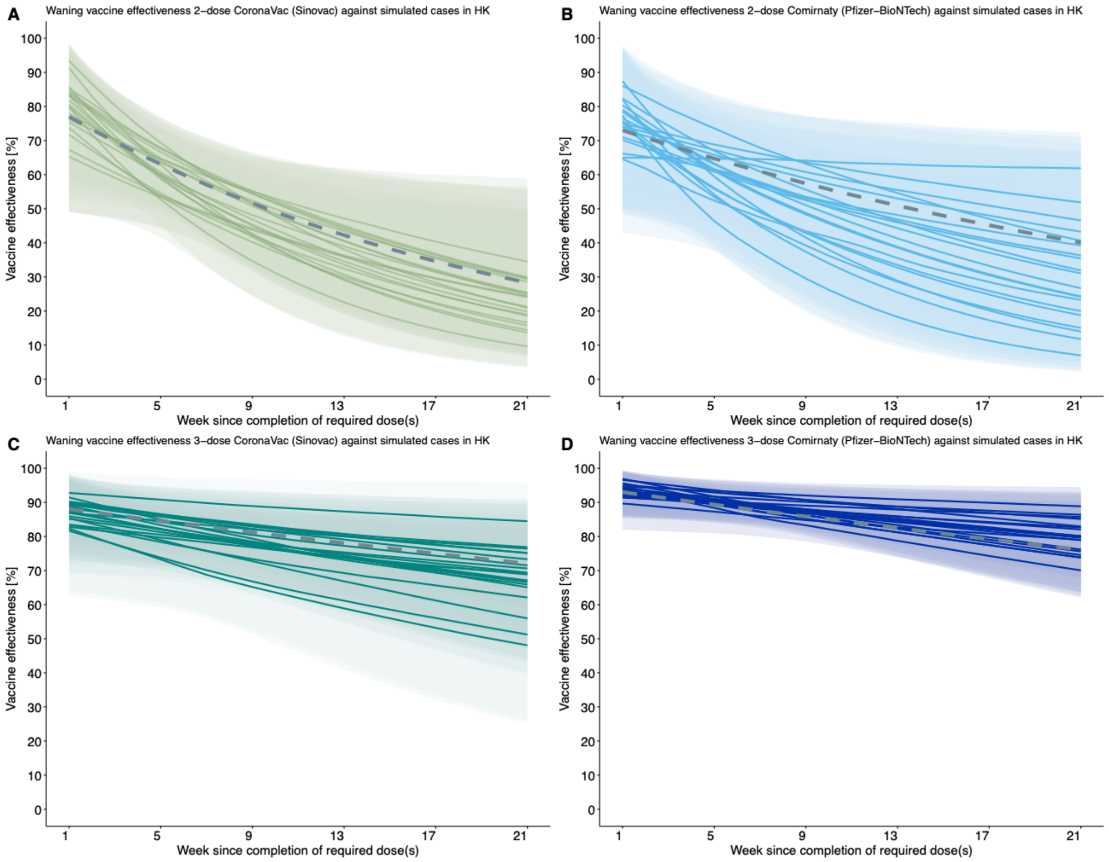

Figure S2. Estimated waning VE through exponential decay using 20 simulated datasets. (A)-(D) The estimated waning effectiveness of CoronaVac and Comirnaty against 20 simulated datasets. The lines indicate the estimation results, with each line representing one simulation. The shaded areas indicate the 95% confidence intervals across simulations. The silver dotted lines are the ground truth vaccine effectiveness.

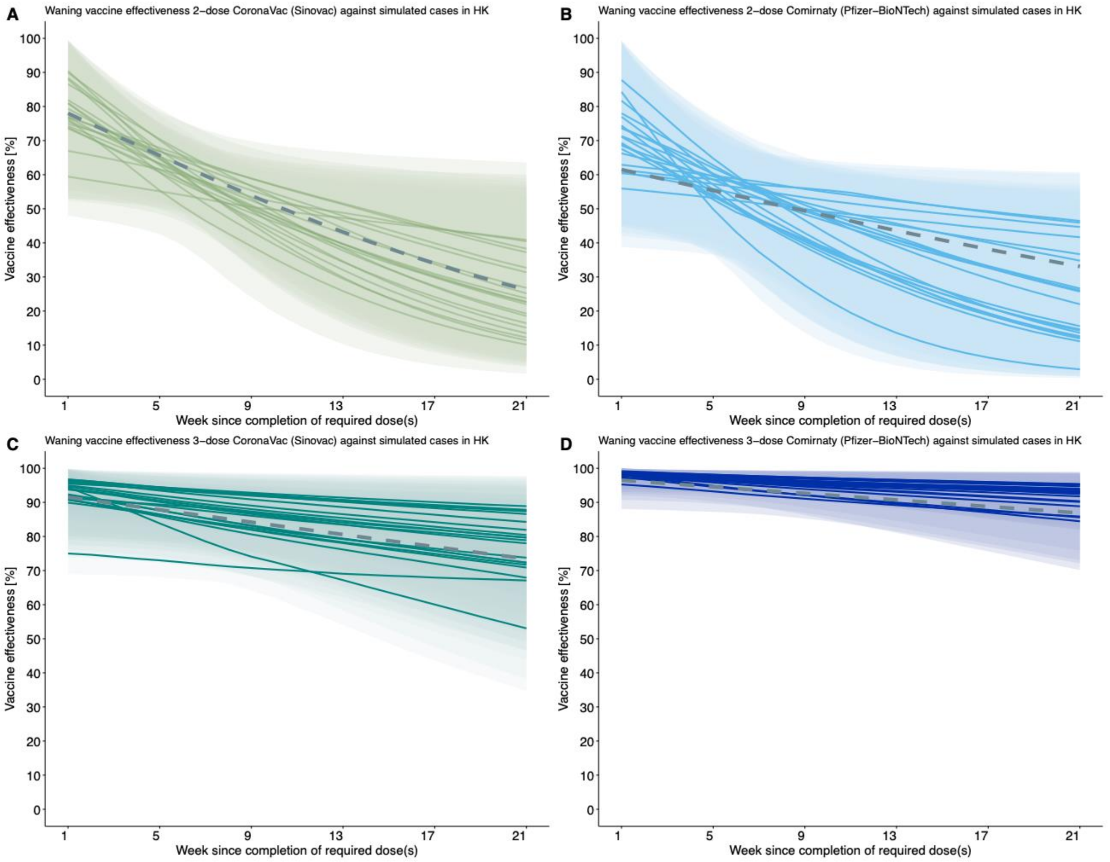

Figure S3. Estimated waning VE through logistic decay using 20 simulated datasets. (A)-(D) The estimated waning effectiveness of CoronaVac and Comirnaty against 20 simulated datasets. The lines indicate the estimation results, with each line representing one simulation. The shaded areas indicate the 95% confidence intervals across simulations. The silver dotted lines are the ground truth vaccine effectiveness.

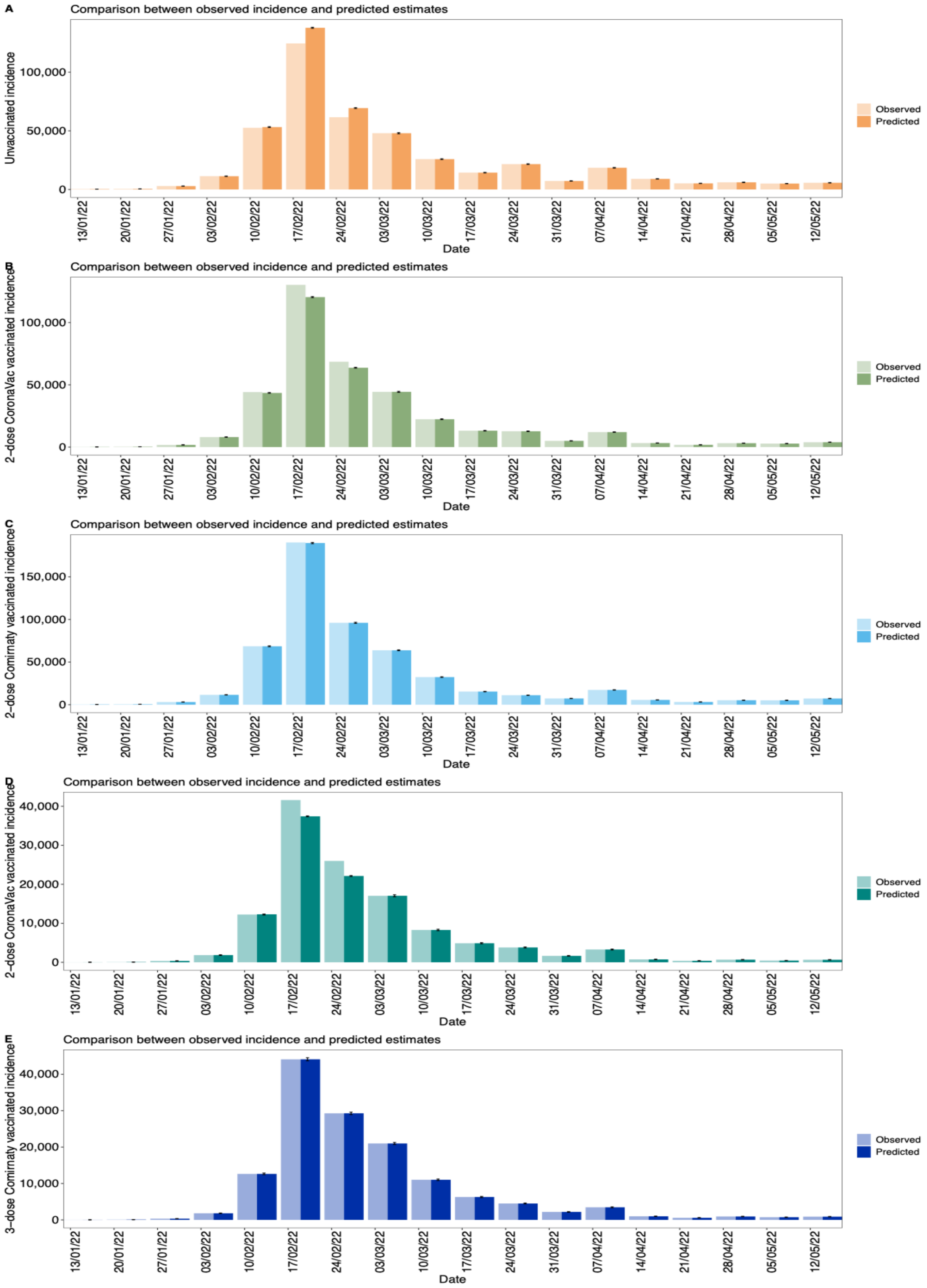

Figure S4. Fitness checking of the exponential decay model with outbreak data in HK through exponential decay model. (A)-(E) Comparison between weekly observed incidence and predicted estimates. The dark bars represent the observed weekly incidence, while the light bars depict the estimated incidence derived from posterior MCMC samples.

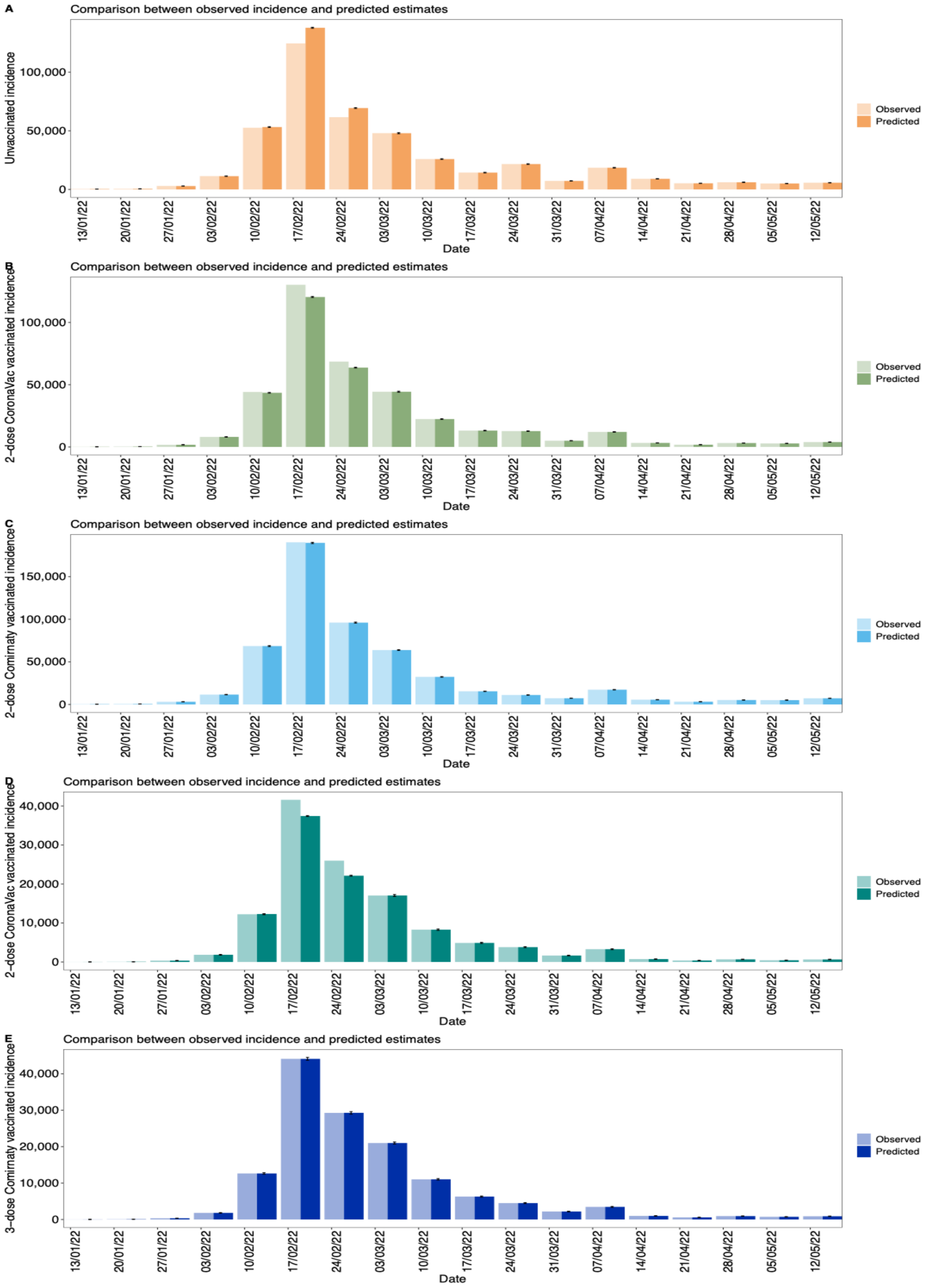

Figure S5. Fitness checking of the logistic decay model with outbreak data in HK through exponential decay model. (A)-(E) Comparison between weekly observed incidence and predicted estimates. The dark bars represent the observed weekly incidence, while the light bars depict the estimated incidence derived from posterior MCMC samples.
